# DFT Study of 1,4-Diazonium-3,6-diolates: Monocyclic Representatives of Unexplored Semi-Conjugated Heterocyclic Mesomeric Betaines

**DOI:** 10.1021/acs.joc.3c00225

**Published:** 2023-05-24

**Authors:** Christopher A. Ramsden, Wojciech P. Oziminski

**Affiliations:** † Lennard-Jones Laboratories, School of Physical and Geographical Sciences, Keele University, Newcastle-under-Lyme, Staffordshire ST5 5BG, U.K.; ‡ Department of Organic and Physical Chemistry, Faculty of Pharmacy, Medical University of Warsaw, 1 Banacha Street, 02-097 Warsaw, Poland

## Abstract

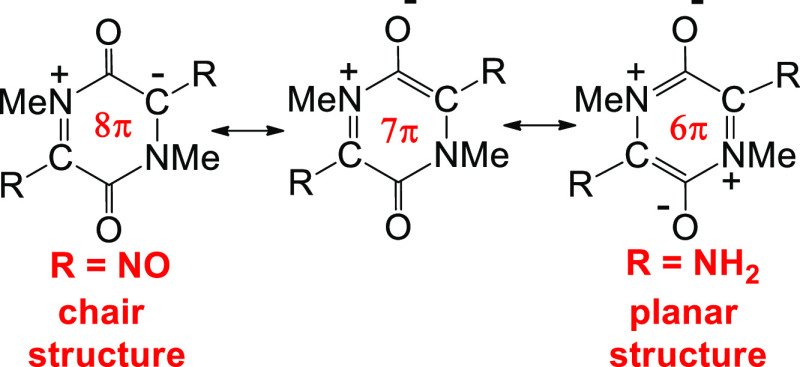

Compared to the well-known conjugated (1,3-dipolar) and cross-conjugated (1,4-dipolar) heterocyclic mesomeric betaines (HMBs), semi-conjugated HMBs are unexplored and almost unknown. The three discrete classes of HMB are defined by the connectivity between their ring 2π heteroatoms and the odd-conjugated fragments that complete the ring. A single example of a stable, fully-characterized semi-conjugate HMB has been reported. This study employs the density functional theory (DFT) methodology to investigate the properties of a series of six-membered semi-conjugated HMBs. The electronic character of ring substituents is found to significantly influence the structure and electronic properties of the ring. The aromaticity measured by HOMA and NICS(1)_zz_ indices is increased by π-electron-donating substituents whereas π-electron-withdrawing substituents decrease the calculated aromatic character and ultimately lead to non-planar boat or chair structures. A notable property of all derivatives is the small energy gap between their frontier orbitals.

## 1 Introduction

**Scheme 1:**
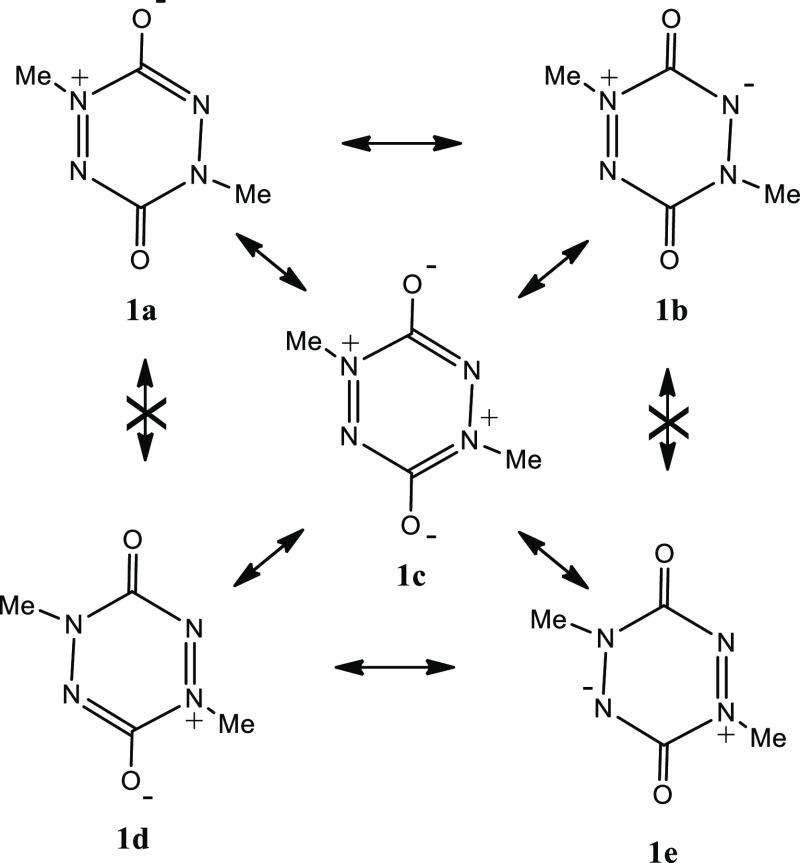
Inter-Relationship of Resonance Forms of Structure **1**

To gain a better understanding of the structure and unusual electronic properties of semi-conjugated heterocyclic mesomeric betaines (HMBs), a neglected class of heterocycles whose chemistry remains unexplored, we describe the results of a density functional theory (DFT) study of simple monocyclic representatives. Three distinct classes of HMB have been recognized: (i) conjugated, (ii) cross-conjugated, and (iii) semi-conjugated, and these can be divided into subgroups depending upon the relative positions of the heteroatoms.^1,2^ The first two classes have been widely explored and are characterized by participation in 1,3-dipolar and 1,4-dipolar cycloadditions, respectively. Semi-conjugated HMBs have only recently been recognized as a smaller but discrete class,^2^ and their properties have received little attention. As far as we are aware, the only example that has been characterized is the 3,6-dioxo-1,2,4,5-tetrazinium derivative **1**, obtained by Neugebauer and co-workers as dark blue plates (mp 146 °C).^3^ The crystal structure of betaine **1** shows a planar molecule with high symmetry. In terms of resonance theory, an unusual feature of semi-conjugated betaine structures is that equivalent resonance structures (e.g., **1a–1d** and **1b–1e**) cannot be interconverted by a simple curly-arrow resonance of the π-electrons (Scheme 1). A tetrapolar form (e.g., **1c**) can be invoked as a “go-between”, but this structure is not consistent with the observed geometry obtained by X-ray analysis. Neugebauer and co-workers observed that the short C–O (1.215 Å) and N–N (1.310 Å) bond lengths are more consistent with a quinonoid structure (i.e., **1b** in resonance with **1e**).

**Figure 1:**
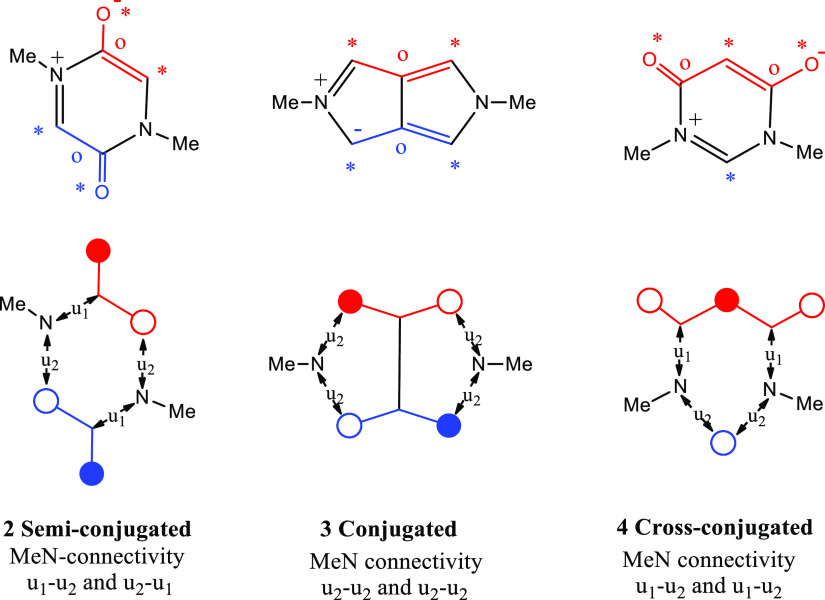
An illustration of the three fundamentally different modes of connectivity between 2π-heteroatoms and associated odd-conjugated fragments in heterocyclic mesomeric betaines.

In terms of molecular orbital theory, the unusual properties of semi-conjugated HMBs arise from the unique connectivity between the 2π-heteroatoms (i.e., contributing two π-electrons, e.g., pyrrole-like NMe) and the odd alternant conjugated π fragments,^4^ e.g., Ṅ–C=O, to which they are connected. For example, in the case of the semi-conjugated heterocycle **2** (Figure 1), the ring is conceptually formed by the union (←u→) of each NMe nitrogen with (i) a non-bonding molecular orbital (NBMO) nodal position (i.e., an unstarred atom °) (←u_1_→) of *one odd alternant fragment* and (ii) a non-nodal NBMO position (i.e., a starred atom *) (←u_2_→) of the *other odd alternant fragment* (Figure 1). This mode of union of the contributing 2π-heteroatoms and odd-conjugated fragments is quite different from the defining connectivities of conjugated, e.g., **3**, and cross-conjugated, e.g., **4**, HMBs.^5^ The structures **3** and **4** are given as representative examples and further examples, together with a description of their subgroups, can be found elsewhere.^1,2,5^ The subtle difference between the connectivities in the semi-conjugated and cross-conjugated betaines (**2** and **4**) should be noted.

The relationship between 2π-heteroatom connectivity and the classification of HMBs as conjugated, cross-conjugated, or semi-conjugated has been discussed in detail in recent reviews.^1,2^ Arising from their novel bonding character, the properties of semi-conjugated mesomeric betaines may have potential applications in new materials. Blue color is relatively rare among organic compounds and, as in azulene, is associated with a small HOMO–LUMO gap and their different spatial arrangement. In the case of azulene, these orbital properties have led to an interest in applications in optoelectronics and other molecular devices.^6^ To obtain a better understanding of this class of heterocyclic mesomeric betaine, we describe here a DFT study of semi-conjugated heterocycles of the general types **5** and **6.**
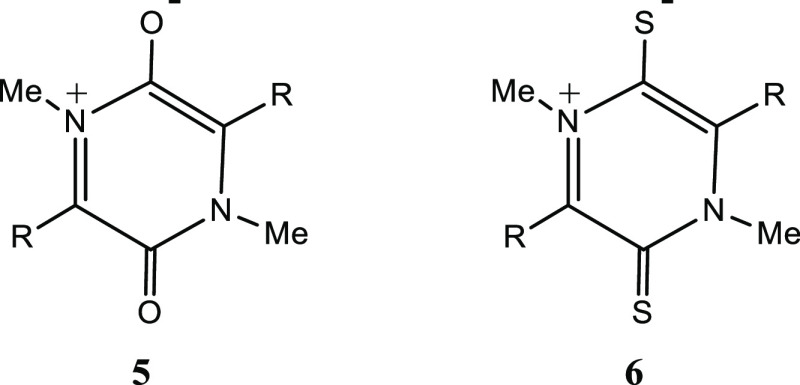


## 2 Results and Discussion

### 2.1 Structure and Substituent Effects

We have calculated the energies, geometries, aromaticity indexes, and frontier orbitals of representative 3,6-dioxo semi-conjugated derivatives **5** (Table 1) and 3,6-dithio derivatives **6** (Table 2) at the B3LYP/6-311++G(d,p) level of theory. Initially, the structures of ten dioxo derivatives **5a–j**, in which the properties of the substituents R are widely varied, were investigated. The calculated bond lengths and angles are shown in Table 1. Apart from the 2,5-dinitroso derivative **5h**, all the derivatives are symmetrical and planar, or close to planarity, as measured by the dihedral angle O–C3–C2–N1 (Table 1). The distortion of the dinitroso derivative (O–C3–C2–N1 167.6°) to a chair form **5h_chair_** is an exception and for comparison we calculated the properties of the corresponding planar structure **5h_planar_**. The Gibbs free energy difference between **5h_chair_** and **5h_planar_** was calculated to be 6.6 kcal mol^–1^ (Table 3).

**Table 1:**
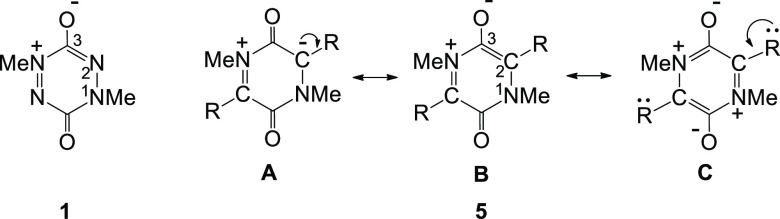
DFT Calculated Gibbs Free Energies and Geometries of HMBs **1** and **5a–k**

entry	structure	CR	*G* (Hartree)	bond lengths (Å)				bond angles (°)		*F*_R_	*R*_R_
				N4–C3	C2–C3	N1–C2	C3–O	C2–C3–N4	O–C3–C2–N1		
1	**1_twist_**	N	–525.479959	1.425	1.370	1.305	1.218	114.2	177.6		
2	**1_planar_[1]**^a^		–525.476659	1.425	1.370	1.305	1.218	114.3	180.0		
	**1_*planar*_**^b^		*–525.246222*	*1.411*	*1.362*	*1.296*	*1.214*	*114.0*	*180.0*		
3	**1_X-ray_**			1.390	1.360	1.310	1.220	115.7	177.9		
4	**5a**	C–H	493.417313	1.420	1.418	1.346	1.236	112.5	180.0	0.0	0.0
	***5a***^b^		*–493.181281*	*1.407*	*1.412*	*1.340*	*1.232*	*112.4*	*180.0*		
5	**5b**	C–CN	677.941116	1.416	1.442	1.359	1.223	113.6	180.0	0.51	0.19
6	**5c**	C–Cl	1412.668368	1.428	1.427	1.352	1.229	112.4	180.0	0.41	0.15
7	**5d**	C–Me	572.025043	1.417	1.426	1.357	1.242	114.2	177.7	0.04	0.13
8	**5e**	C–F	691.955899	1.429	1.416	1.343	1.230	110.7	180.0	0.43	0.34
9	**5f**	C–OH	643.925697	1.402	1.415	1.349	1.251	114.2	180.0	0.29	0.64
	***5f***^b^		*–643.652315*	*1.390*	*1.408*	*1.341*	*1.247*	*114.2*	*180.0*		
10	**5g**	C–NH_2_	604.152609	1.406	1.420	1.360	1.249	114.4	180.0	0.02	0.68
11	**5h_chair_**	C–NO	752.060075	1.421	1.459	1.360	1.215	114.6	167.6		
12	**5h_planar_[5]**^a^		752.049581	1.436	1.463	1.351	1.213	115.2	180.0	0.50	0.45
	***5h*_*planar*_**^b^		*–751.72468*	*1.422*	*1.450*	*1.346*	*1.212*	*114.9*	*180.0*		
13	**5i**	C–CF_3_	1169.676519	1.432	1.434	1.353	1.225	113.4	173.0	0.38	0.19
14	**5j**	C–NO_2_	902.500813	1.430	1.429	1.342	1.222	111.5	178.3	0.67	0.16
15	**5k_boat_**	C–SO_2_F	1789.197965	1.436	1.436	1.384	1.219	112.8	167.2		
16	**5k_planar_[3]**^a^		1789.187972	1.442	1.434	1.350	1.219	112.3	180.0	0.75	0.22

a Number of imaginary frequencies of a planar structure.

b Structures optimized at the CAM-B3LYP/6-311++G(d,p) level of theory.

**Table 2:**
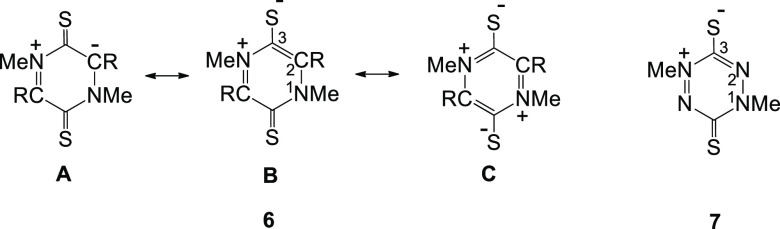
DFT Calculated Gibbs Free Energies and Geometries of HMBs **6a–d** and **7**

entry	structure	CR	*G* (Hartree)	bond lengths (Å)				bond angles (°)		*F*_R_	*R*_R_
				N4–C3	C2–C3	N1–C2	C3–S	C2–C3–N4	S–C3–C2–N1		
1	**6a**	C–H	–1139.352549	1.408	1.403	1.342	1.692	113.2	180.0	0.0	0.0
2	**6b**	C–F	–1337.889730	1.415	1.403	1.341	1.684	112.3	180.0	0.43	–0.34
3	**6c_chair_**	C–NO	–1397.989630	1.385	1.433	1.368	1.671	116.5	–165.0		
4	**6c_planar_[5]**^a^		–1397.956854	1.430	1.443	1.351	1.669	114.6	180.0	0.50	0.45
5	**6d_boat_**	C–SO_2_F	–2435.111661	1.418	1.419	1.348	1.670	113.3	–152.0		
6	**6d_planar_[3]**^a^		–2435.088639	1.443	1.418	1.348	1.677	112.4	180.0	0.75	0.22
7	**7_boat_**	N	–1171.399368	1.413	1.355	1.304	1.667	113.4	–163.9		
8	**7_planar_[1]**^a^		–1171.396573	1.421	1.355	1.299	1.670	114.2	180.0		

a Number of imaginary frequencies of planar structure

**Table 3:**
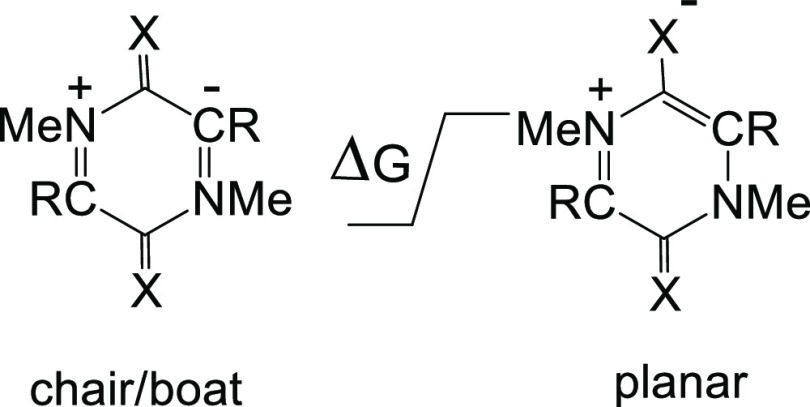
DFT Calculated Gibbs Free Energy Differences (Δ*G*) of Planar and Non-planar HMBs

entry	structure	X	CR	Δ*G* (kcal/mol)
1	**5h**	O	C–NO	6.6
2	**5k**	O	C–SO_2_F	6.3
3	**6c**	S	C–NO	20.6
4	**6d**	S	C–SO_2_F	14.4
5	**1**	O	N	2.1
6	**7**	S	N	1.8

In view of the substantial charge separation in the studied compounds, to validate the reliability of the B3LYP functional, we performed additional geometry optimization using the long-range corrected CAM-B3LYP functional with the same basis set for the selected molecules **1_planar_, 5a**, **5f** and **5h_planar_**. The results, shown in Table 1, indicate that the CAM-B3LYP optimized bond lengths differ only by an order of 0.01 Å from the B3LYP optimized structures. Angle differences are smaller than 0.5 degrees, and there are no dihedral angle differences. We can conclude that the geometries provided by both DFT methods are very similar.

To verify which DFT methods yield geometries that are closer to the CCSD(T) ideal geometries, we performed single point CCSD(T) calculations on these two geometries, i.e., B3LYP and CAM-B3LYP for the selected molecules **1_planar_, 5a**, **5f**, and **5h_planar_**. The calculated energies, denoted as CCSD(T)//DFT, are shown in Table 4. These results show that for all the selected molecules the CCSD(T) energy is lower for the B3LYP geometry Therefore, we conclude that the B3LYP method gives a geometry that is closer to the ideal CCSD(T) geometry than the CAM-B3LYP method, and for the rest of the study we have used only the B3LYP method.

**Table 4:** Absolute and Relative Values of Single Point Energy Calculated at the CCSD(T)/6-311++G(d,p) Level on Structures Optimized at B3LYP/6-311++G(d,p) and CAM-B3LYP/6-311++G(d,p) Levels of Theory

structure	*E*(CCSD(T)//B3LYP) (Hartree)	*E*(CCSD(T)//CAM-B3LYP) (Hartree)	Δ*E* (kcal/mol)
**1_planar_**	–524.2757257	–524.2741018	1.02
**5a**	–492.2792871	–492.2781307	0.73
**5f**	–642.4713257	–642.4700108	0.83
**5h_planar_**	–750.3384139	–750.3360975	1.45

Inspection of Table 1 shows the variation of bond lengths with the nature of the substituents R. A multiple linear analysis of the O–C3 bond lengths of the derivatives **5a-j** (Entries 4-10,12-14), using **5h_planar_** (Entry 12), revealed the following significant relationship (eq 1) between C3–O and the Swain and Lupton electronic substituent constants *F*_R_ and *R*_R_ of the substituents R.^7^
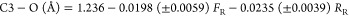
1
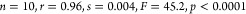


We interpret this correlation to mean that substituents R with positive values of *F*_R_ and *R*_R_ will favor resonance forms of the type **5A** (Table 1) with consequent shortening of the C–O bonds, and there is the corresponding lengthening of the C–C and N–C bonds, as illustrated by the bond lengths shown in Table 1. Presumably, in the extreme case of R = NO (*F*_R_ + *R*_R_ = 0.95), the electron distribution **5A** and associated bond lengths disfavor the planar structure, and a non-planar chair form is more stable.

Substituents with large positive values of both *F*_R_ and *R*_R_ are uncommon. To test the correlation (eq 1) and to further explore the influence of *F*_R_ and *R*_R_ on ring geometry, we calculated the properties of the derivative **5k** (R = SO_2_F, *F*_R_ + *R*_R_ = 1.00). As for derivative **5h**, the minimum energy corresponds to a non-planar structure; in this case, the ring distorted to a boat form **5k_boat_**. The energy difference relative to the planar form **5k_planar_** was calculated to be 6.3 kcal mol^–1^, which is close to the difference calculated for **5h** (6.6 kcal mol^–1^) (Table 3, Entries 1 and 2).

Table 5 shows the DFT/B3LYP calculated charge distribution on the ring and exocyclic oxygen atoms for structures **1** and **5a–k**. As expected the charge on oxygen increases as the C–O bond length increases (compare **5a** and **5f**). Shortening of the C–O bond is associated with a lower negative charge on oxygen (compare **5a** and **5f**). The positive charge is associated with the ring carbon atoms (C2 and C3), but the relative distribution depends on the substituent R, particularly at C2. The more electronegative the substituent, the more positive charge on C2 (compare: **5g**, **5f**, **5e**). The charge on the nitrogen ring atoms is made more negative than in **5a** by pi-donors and sigma-acceptors (**5e**,**5f**,**5g**).

**Table 5:** DFT calculated NPA Atomic Charges of HMBs **1** and **5a–k**

structure	N/CR	natural atomic charge			
		N1	C2	C3	O
**1_twist_**	N	–0.153	–0.228	0.689	–0.625
**1_planar_**		–0.152	–0.228	0.689	–0.625
**5a**	C–H	–0.359	0.002	0.526	–0.674
**5b**	C–CN	–0.342	0.052	0.583	–0.618
**5c**	C–Cl	–0.393	0.120	0.528	–0.653
**5d**	C–Me	–0.370	0.181	0.538	–0.698
**5e**	C–F	–0.401	0.562	0.489	–0.649
**5f**	C–OH	–0.386	0.467	0.487	–0.718
**5g**	C–NH_2_	–0.392	0.320	0.516	–0.721
**5h_chair_**	C–NO	–0.384	0.258	0.599	–0.561
**5h_planar_**		–0.359	0.279	0.581	–0.569
**5i**	C–CF_3_	–0.345	0.078	0.556	–0.632
**5j**	C–NO_2_	–0.365	0.289	0.547	–0.615
**5k_boat_**	C–SO_2_F	–0.364	–0.063	0.551	–0.603
**5k_planar_**		–0.369	–0.054	0.549	–0.611

It is interesting to compare the DFT calculated structure of the 1,2,4,5-tetrazinium derivative **1** with the X-ray structure. Derivative **1** is reported to be planar, but inspection of the published data^3^ reveals that there is a slight distortion from planarity; the torsion angle O–C3–C2–N1 is 177.9°. The calculated structure has a similar small distortion (O–C3–C2–N1 is 177.6°). The calculated energy difference between distorted structure **1_twist_** and fully planar **1_planar_** is 2.1 kcal mol^–1^ (Table 3, Entry 5). Based on the observed electronic effects of NO and SO_2_F substituents, it might be expected that the ring N atoms at positions 2 and 5 would result in greater distortion from planarity, but this is not the case.

Properties of the five 3,6-dithio derivatives **6a–d** and **7** are summarized in Table 2. The parent derivative **6a** and the fluoro derivative **6b** are both symmetrical and planar. However, the NO derivative **6c** and the SO_2_F derivative **6d** have non-planar energy minima with chair/boat structures (Table 2, Entries 3 and 5) similar to the corresponding 3,6-dioxo derivatives **5h** and **5k**, and presumably due to similar substituent effects. The calculated energy differences **6c_chair_**/**6c_planar_** (20.6 kcal mol^–1^) and **6d_boat_**/**6d_planar_** (14.5 kcal mol^–1^) (Table 3, Entries 3 and 4) are larger than those for the corresponding 3,6-dioxo derivatives (Table 3, Entries 1 and 2). It is interesting to note that the 3,6-dithiotetrazinium derivative **7** adopts a boat configuration (**7_boat_**; S–C3–C2–N1 −163.9°)(Table 2, Entry 7) with shortening of the C–S bonds relative to **6a**. This contrasts with the slight twist of the 3,6-dioxo analogue **1_twist_**, but the energy differences **7_boat_**/**7_planar_** and **1_twist_**/**1_planar_** are similar (Table 3, Entries 5 and 6).

### 2.2 Aromaticity and Bonding

In a previous study,^8^ we have calculated the aromatic stabilization energies (ASE) of the derivatives **1** and **5a** using homodesmotic schemes. These ASE values (**1**, 6.5 kcal mole^–1^; **5a**, 18.1 kcal mole^–1^) suggest that cyclic conjugation makes a positive contribution to stabilizing these heterocyclic rings. Since derivative **1** has been isolated as a stable solid, these results suggest that **5a**, with a significantly greater ASE value, might be a stable ring.

The Harmonic Oscillator Model of Aromaticity (HOMA) index is a geometry-based index of classical aromaticity.^9,10^ Calculated HOMA values for planar derivatives **5a–k** are shown in Table 5. The values for rings **1** and **5a** (Table 6, Entries 1 and 3) are similar and show a modest degree of classical aromaticity. The values for benzene and pyridine are 1.0 and 0.99, respectively. The HOMA values for derivatives **5a–k** are in the range 0.19–0.79 (Table 6, Entries 3–12) and there is a correlation between HOMA and the Swain and Lupton electronic resonance constant *R*_R_, which accounts for over 70% of the variation (HOMA = O.549–0.378 *R*_R_, *r* = 0.85, *s* = 0.09, *F* = 23.2, *p* = 0.0009). The inclusion of an *F*_R_ term does not significantly improve the correlation. Substituents with negative *R*_R_ values, e.g., NH_2_, OH, have higher aromaticity.

**Table 6:** DFT Calculated Aromaticity Indices and Orbital Properties for HMBs **1**, **5** and Azulene

entry	structure	CR	HOMA	NICS(1)_ZZ_	pEDA	HOMO^a^	LUMO^a^	FMO gap	VIP^a^	VEA^a^
1	**1_twist_**	N	0.70	–7.6	0.907	–6.485	–4.158	2.327		
2	**1_planar_**		0.70	–7.2	0.905	–6.484	–4.164	2.320	8.51	2.17
3	**5a**	C–H	0.68	–10.4	0.808	–5.351	–2.813	2.539	7.30	0.94
4	**5b**	C–CN	0.52	–7.5	0.857	–6.591	–4.480	2.040	8.27	2.77
5	**5c**	C–Cl	0.59	–8.4	1.025	–5.564	–3.135	2.430	7.36	1.39
6	**5d**	C–Me	0.64	–9.3	0.811	–4.996	–2.471	2.520	6.82	0.71
7	**5e**	C–F	0.65	–9.9	0.973	–5.596	–3.009	2.590	7.55	1.09
8	**5f**	C–OH	0.79	–10.6	0.997	–5.014	–2.317	2.700	6.93	0.45
9	**5g**	C–NH_2_	0.73	–10.6	1.038	–4.462	–1.898	2.560	6.29	0.13
10	**5h_chair_**	C–NO				–6.649	–5.201	1.450		
11	**5h_planar_**		0.19	–4.0	0.630	–6.760	–5.330	1.430	8.76	3.59
12	**5i**	C–CF_3_	0.51	–6.4	0.833	–6.263	–4.025	2.240	8.10	2.25
13	**5j**	C–NO_2_	0.57	–7.8	0.880	–6.590	–4.454	2.140	8.37	2.75
14	**5k_boat_**	C–SO_2_F				–6.890	–4.781	2.110		
15	**5k_planar_**		0.45	–6.0	0.925	–6.809	–4.634	2.170	8.54	2.97
16	**azulene**					–5.558	–2.269	3.288		

a Electron volts (eV).

The Nucleus-Independent Chemical Shifts (NICS(1)_zz_) index is a measure of magnetic aromaticity and more negative values indicate higher aromaticity.^11^ Using the B3LYP/6-311++G(d,p) method, the value for benzene is −29.76. The NICS(1)_zz_ values for derivatives **5b-k** are in the range −4.05 to −10.65 (Table 6, Entries 4–15), and there is also a significant positive correlation with the resonance constant *R*_R_ (NICS(1)_zz_ = −7.918 + 5.129 *R*_R_, *r* = 0.86, *s* = 1.15, *F* = 26.5, *p* = 0.0006). In this case, the contribution of the resonance parameter *R*_R_ is also dominant and a contribution of *F*_R_ makes little improvement to the correlation.

**Figure 2:**
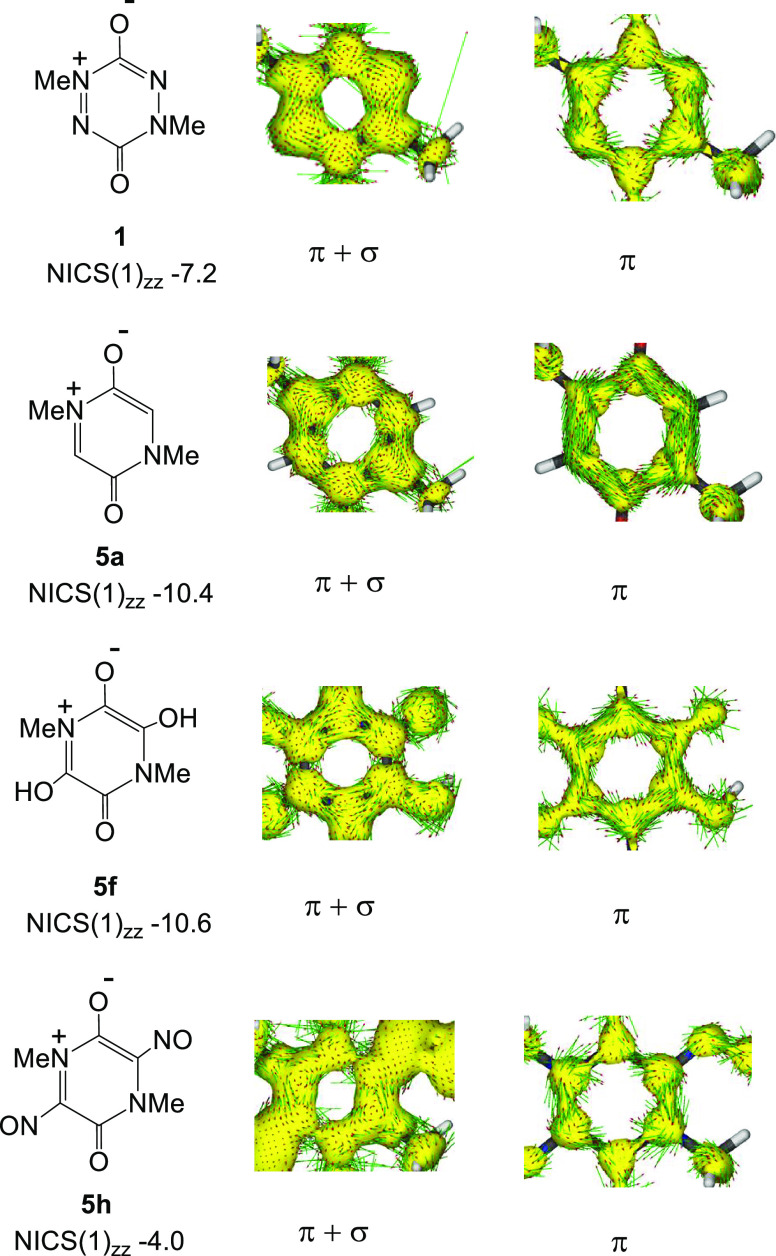
ACID π + σ and π electron current density maps for rings **1**, **5a**, **5f**, and **5h_planar_**.

Although NICS values have been recorded for a wide variety of heterocyclic rings, their use without accompanying inspection of current density maps to authenticate the existence of a ring current has been criticized.^12,13^ We have therefore used the anisotropy of induced current density (ACID) method^14^ to investigate electron delocalization in the rings **1**, **5a**, **5f**, and **5h**. The observed σ + π and π electron current density maps are shown in Figure 2. The π electron maps for rings **5a** and **5f** are consistent with the moderate π cyclic conjugation suggested by their NICS(1)_zz_ values (−10.4 and −10.6, respectively). Conjugation for the diaza derivative **1** appears to be weaker, which is consistent with a lower NICS(1)_zz_ value (−7.2), and the disrupted conjugation in the nitroso derivative **5h_planar_** reflects the much lower NICS(1)_zz_ value (−4.0). We conclude that the NICS(1)_zz_ values do reflect the magnitude of the π electron ring currents in this class of heterocycle and that the rings **1** and **5a** are associated with moderate magnetic aromaticity. It may be significant that the derivative **5h_planar_,** which distorts to a non-planar form, has a π electron map (Figure 2) that resembles the antiaromatic resonance form **5A** (Table 1) rather than the aromatic form **5C**.

According to the HOMA and NICS(1)_zz_ indexes (Table 6), the heterocycles **5** have moderate to weak aromatic character. For heterocyclic rings, the variation of classical and magnetic aromaticity with structure can be notably different,^15^ but for the derivatives **5** there appears to be a common variation with the resonance *R*_R_ parameter of the substituents R. The index pEDA (pi Electron Donor Acceptor) is a measure of the π population and is the sum of the *p_z_* atomic orbital population minus the aromatic sextet value of six. Calculated pEDA values for the planar rings **5a-k** are shown in Table 6 and are in the range 0.6–1.1. With an average pEDA value of 0.8, this is consistent with the 7π representation **5**B and its symmetry-related hybrid.

The property that dominates the variation in aromaticity is the resonance constant *R*_R_ of the substituents R. In terms of resonance theory, we interpret this to mean that π-electron-donating substituents R with negative values of *R*_R_, e.g., OH and NH_2_, favor a greater contribution from the tetrapolar aromatic sextet structure **5**C (Table 1) with the negative charge located on the exocyclic oxygen atoms. This is consistent with greater calculated aromaticity, the observed increase in C–O bond lengths and the decrease in the C3–N4 bond lengths (eq 1). In contrast, for derivatives having substituents R with a high positive value of *R*_R_, e.g., NO and SO_2_F, the contribution from the antiaromatic 8π hybrid **5**A is increased with a consequent shortening of the O–C3 bonds. In extreme cases, this results in a non-planar boat or chair structure being more stable (Table 3). The effects of *R*_R_ on structure are augmented by field effects *F*_R_, which are in the range 0–0.75 and will favor contributions from the hybrid **5**A but these field effects appear to be small or insignificant for the aromaticity indices HOMA and NICS(1)_zz_.

It seems counter intuitive that an increase of π electrons (pEDA) in the electron-rich rings **5** also increases the aromaticity. However, HOMA is a geometry-based index of aromaticity and depends upon the distribution of the electrons, as well as the number. If the ring bond lengths trend toward aromatic lengths as pEDA increases then the HOMA calculated aromaticity will also increase. This may not imply an increase in thermodynamic stability. It should also be noted that the effects of substituents R on pEDA vary according to Eq 2. Thus substituents with a positive *F*_R_ value will increase pEDA by inducing back-donation from the exocyclic oxygens whereas substituents with a positive *R*_R_ value will reduce pEDA by resonance.
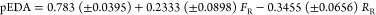
2
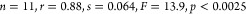


Table 7 shows calculated HOMA and NICS(1)_zz_ values for selected planar sulfur derivatives **6a–d** and **7**. The HOMA and NICS(1)_zz_ results show similar trends to the oxa analogues **5a–k** (Table 4). The values for derivatives **5c** and **5d** (Table 6, Entries 3 and 4) indicate low aromaticity. This is consistent with some contributions from the 8π tautomers **6**A and the preferred chair/boat structures **6c**_chair_ and **6d**_boat_ (Table 3). The NICS(1)_zz_ values for **6c** and **6d** are so low that they are positive, suggesting significant antiaromatic character in the planar form. Interestingly, the diaza derivative **7** (Table 7, Entry 7) is also non-planar and has very low magnetic aromaticity.

**Table 7:** DFT Calculated Aromaticity Indices and Orbital Properties for HMBs **6** and **7**

entry	structure	CR	HOMA	NICS(1)_ZZ_	pEDA	HOMO^a^	LUMO^a^	FMO gap	VIP^a^	VEA^a^
1	**6a**	C–H	0.81	–4.2	0.858	–5.317	–3.261	2.060	7.01	1.59
2	**6b**	C–F	0.77	–6.3	1.088	–5.524	–3.369	2.160	7.22	1.68
3	**6c_chair_**	C–NO				–6.321	–4.804	1.520		
4	**6c_planar_**		0.44	5.3	0.840	–6.217	–5.313	0.900	7.80	3.72
5	**6d_boat_**	C–SO_2_F				–6.385	–4.762	1.620		
6	**6d_planar_**		0.69	3.5	1.022	–6.312	–4.722	1.590	7.89	3.23
7	**7_boat_**	N				–6.037	–4.263	1.770		
8	**7_planar_**		0.75	4.8	1.003	–5.991	–4.439	1.550	7.73	2.72

a Electron volts (eV).

### 2.3 Frontier Orbitals and Reactivity

The kinetic stability of the rings **5** is also of interest. The known derivative **1** has been shown to be reduced to a stable radical anion, indicative of a low energy LUMO and a high electron affinity. Calculated frontier orbital energies (HOMO and LUMO) together with calculated vertical ionization potentials (VIP) and vertical electron affinities (VEA) for derivatives **1** and **5a-k** are shown in Table 6. Again, the substituent effects can be understood by examination of the statistically significant correlations with the substituent constants *F*_R_ and *R*_R_ (Eqs 3–6).
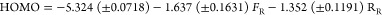
3
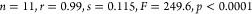

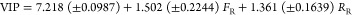
4
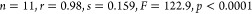

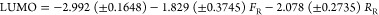
5
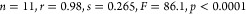

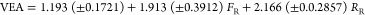
6
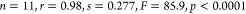


Inspection of eq 3 shows that the HOMO energy is lowered by positive substituent field and resonance effects and, as expected, substituents have a very similar effect on the VIPs (eq 4). For derivatives **5a–k,** the VIPs vary in the range 6.3–8.5 eV. The most aromatic derivatives (with high negative *R*_R_) have the lowest VIPs (6.2–7.5 eV) (Table 6, Entries 7–9); substituents with positive *F*_R_ and *R*_R_ values have VIPs in the range 8.1–8.8 (Table 6, Entries 4,10–14). The derivatives **5** are electron-rich heterocycles. For comparison, the ionization potentials of pyrrole and 2,4-dimethylpyrrole are 8.3 and 7.5 eV, respectively.^16,17^

The calculated LUMOs and VEAs show a similar dependence on substituent *F*_R_ and *R*_R_ values (eqs 5 and 6), but the resonance effect (*R*_R_) makes a slightly greater relative contribution. The VEA values are in the range 0.1–3.6 eV, with the higher values associated with substituents with high positive *R*_R_ values (CN, NO, SO_2_F) (Table 4, Entries 4,10,14).

Comparison of eqs 3 and 5 reveals that the energy gap between HOMO and LUMO (FMO Gap) is greatest for substituents having negative *R*_R_ values (Table 6, Entries 5–9) and smallest when *F*_R_ and *R*_R_ are both large and positive (Table 6, Entry 10). In all the derivatives **5**, the frontier orbital gap is smaller than that for azulene calculated using the same method (Table 6, Entry 16).

**Figure 3:**
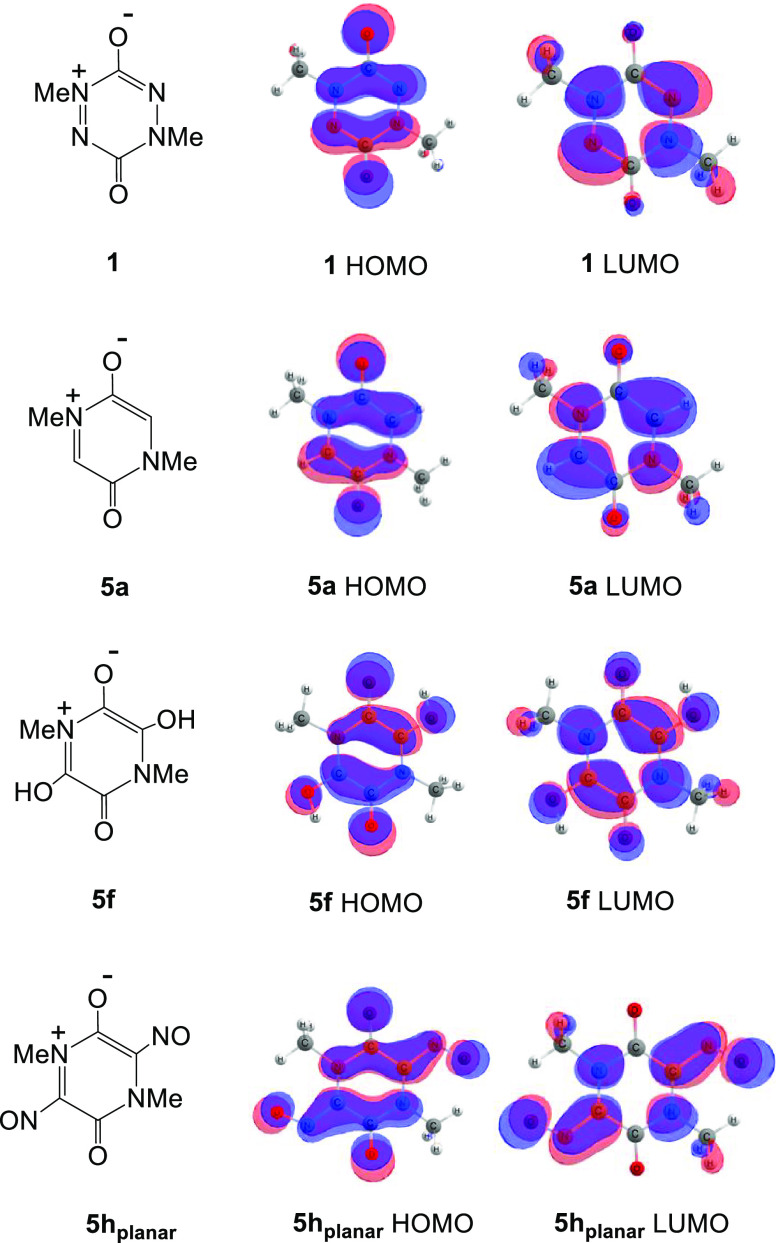
The DFT/B3LYP calculated frontier orbitals of **1** and **5a**, **5f** and **5h**.

Frontier orbital maps for structures **1**, **5a**, **5f**, and **5h** are shown in Figure 3. Inspection shows significant differences in charge distribution in HOMO and LUMO. In particular, there is large electron density on the exocyclic oxygen atoms in the HOMOs but this is considerably or completely transferred to the ring in the LUMOs. This is significant in terms of electronic transitions and color. The energy difference between frontier orbitals is not necessarily an accurate guide to excitation energies since electron repulsion is not satisfactorily accounted for. If the HOMO and LUMO are localized in different areas of space, overlap densities can be small and this leads to an over-estimate of electron repulsion and the HOMO–LUMO energy separation. This is the case for azulene in which charge distribution in the HOMO is predominantly on the five-membered ring, but in the LUMO is on the seven-membered ring. This largely explains why azulene is blue but anthracene is colorless, despite similar HOMO–LUMO gaps.^6b^

Based on the calculated frontier orbital energies (Table 6), the frontier molecular orbital gap (FMO gap) for structure **1** is equal to 2.327 eV. This equates to absorption at a wavelength of 533 nm and a blue or blue-green color. In fact compound 1 is blue-black with absorption at 603 nm (lg ε 3.5). This difference between calculated and observed energies may be related to the different electron distribution in HOMO and LUMO. The colors of other derivatives can be predicted using calculated FMO gaps (Tables 6 and 7).

Table 7 shows frontier orbital properties for five dithio derivatives **6a–e**. Comparison with the corresponding dioxa derivatives **5** shows that these have greater ring π density (pEDA), slightly higher HOMOs, lower LUMOs, and smaller frontier orbital gaps (FMO Gap). Correspondingly, the VIPs are smaller (7.0–7.9 eV) and the VEAs bigger (1.6–3.2 eV).

## 3 Conclusions

The planar 3,6-dioxa heterocycles **5** have an interesting electronic profile. The resonance structures **5B** suggest they are 7π ring systems and this is broadly consistent with their pEDA values. Aromaticity indices (HOMA and NICS(1)_zz_) suggest that they are weakly aromatic with little evidence of antiaromaticity. This is consistent with the calculated ASE (18.1 kcal mole^–1^) of the unsubstituted derivative **5a**. Substituents at the 2 and 5 positions influence the properties. Substituents with a negative resonance effect (e.g., Cl, Me, F, OH, and NH_2_) increase the ring π population but also increase the aromaticity indices values. This effect can be attributed to the influence on ring bond lengths resulting in a greater contribution from the 6π resonance structures **5C**. In contrast, substituents with positive resonance effects and large field effects appear to increase the contribution of the antiaromatic 8π resonance structures **5A**. Accordingly, the derivatives **5h,k** (R = NO, SO_2_F) have lower aromaticity indices and favor non-planar boat/chair structures. Rings **5** are electron-rich, as measured by HOMO energy and VIP and have small frontier orbital gaps (FMO Gap) in the range 2.1–2.7 eV, which is smaller than that in azulene (3.2 eV). The VEA values are low suggesting the ready formation of a radical anion.

The calculated properties suggest that stable derivatives of the planar structures **5** may be accessible. In terms of stability and reactivity, the 1,2,4,5-tetrazinium derivative **1** is the only bench mark for the heterocycles **5**; it is stable, crystalline, and can be reduced to a radical anion. The calculated properties of derivative **1** are in good agreement with its structure (Table 1) and reactivity. It appears to have modest aromaticity, as measured by HOMA and NICS(1)_zz_ indices, can be expected to be not easily oxidized (VIP 8.51 eV) but susceptible to reduction (VEA 2.17 EV). The derivatives that come closest in properties to derivative **1** are **5a** (R = H), **5e** (R = F) and **5i** (R = CF_3_). The unsubstituted **5a** and 2,5-difluoro **5e** derivatives have similar aromaticity profiles as the 1,2,4,5-tetrazinium derivative **1**, with a slightly higher VIPs (7.30 and 7.55 eV), making them easier to oxidize, but much smaller VEAs (0.94 and 1.09 eV), making them more stable to reduction. The trifluoromethyl derivative **5i** has a VIP (8.10 eV) closer to that of **1** but the VEA (2.25) is higher. Ring nitrogen often has a similar effect to a nitro substituent, e.g., pyridine and nitrobenzene. The 2,5-dinitro derivative **5j** does have a similar profile to the tetrazinium derivative **1** except that the VEA (2.75 eV) is significantly higher making it more vulnerable to reduction.

The semi-conjugated HMBs, typified by the monocyclic derivatives **5** and **6**, occupy an area of heterocyclic chemistry that is virtually unexplored. Their unusual structural properties and electronic profile, including a small frontier orbital separation, merit further investigation, including synthetic studies. Unfortunately, they are not natural product but, nevertheless, they present a challenge that may be rewarding.

## 4 Computational Details

All calculations were performed using the Gaussian 16 suite of programs.^18^ The hybrid functional B3LYP^19,20^ was used in conjunction with triple-zeta Pople basis set 6-311++G(d,p).^21,22^ For selected molecules: **1_planar_**, **5a**, **5f**, and **5h_planar,_** additional geometry optimizations with CAM-B3LYP^23^ functional were performed, followed by single point energy CCSD(T)^24^ calculations. All geometry optimizations were followed by frequency calculations to establish the nature of the stationary point and to calculate the ZPE and thermal corrections to Gibbs free energy. True energy minima have no imaginary frequencies and the number of imaginary frequencies of structures being saddle points is reported in the text. Vertical ionization potential (VIP) and vertical electron affinity (VEA) were calculated as the energy difference between a neutral molecule and a positive/negative ion with the same molecular geometry.^25^ Three aromaticity indices were calculated—geometric HOMA,^9,10^ magnetic NICS(1)_ZZ_ ,^26,27^ and electronic pEDA.^28,29^ HOMA and pEDA were calculated using the free AromaTcl software.^30^ The NICS(1)_ZZ_ index was calculated as the z-component(perpendicular) of the shielding constant of a ghost atom laying 1 Å above the geometric centre of the ring. ACID maps were calculated by using the software package developed by Herges and Geuenich.^31^ Total atomic charges were calculated according to the NPA (Natural Population Analysis) scheme by the Natural Bond Orbital (NBO) version 3.1 program interfaced to Gaussian.

The multiple regression relationships and associated statistics based on the Ordinary Least Squares method were determined using online software.^32^ The meaning of the statistical parameters are the following: *n* = sample size, *r* = multiple regression correlation coefficient, *s* = residual standard deviation, *F* = Fisher test value, *p* = *p*-value.

## Data Availability

The data underlying this study are available in the published article and its Supporting Information.

## Supporting Information Available

Atom coordinates and absolute energies of calculated structures (PDF)
