# Supplementary material for: DFT Study of 1,4-Diazonium-3,6-diolates: Monocyclic Representatives of Unexplored Semi-Conjugated Heterocyclic Mesomeric Betaines
Source: J Org Chem. 2023 May 24;88(13):8248–56. doi: 10.1021/acs.joc.3c00225 (PMC10337032; doi:10.1021/acs.joc.3c00225)
Supplement: Supplementary file 1 — jo3c00225_si_001.pdf [file jo3c00225_si_001.pdf]

<u>**Supporting Information**</u>

A DFT Study of 1,4-Diazonium-3,6-diolates: Monocyclic Representatives of Unexplored Semi-Conjugated Heterocyclic Mesomeric Betaines.

Christopher A. Ramsden[a]* and Wojciech P. Oziminski[b]*

[a]Lennard-Jones Laboratories, School of Physical and Geographical Sciences, Keele University, Staffordshire ST5 5BG, UK

[b]Department of Organic and Physical Chemistry, Faculty of Pharmacy, Medical University of Warsaw, 1 Banacha Street,02-097 Warsaw, Poland

Table of Contents

Table 1. Structures of compound type **1** S2

Table 2. Structures of compound type **5** S5

Table 3. Structures of compound type **6** S25

Table 4. Structures of compound type **7** S32

Table 1. Structures of compound type 1

1-twist

	angstroms		
	X	Y	Z
C	1.08927800	0.89567800	0.00714100
C	-1.08923200	-0.89564600	0.00897300
N	-0.19318100	1.37690500	-0.03444500
N	-1.18174300	0.52539000	-0.03659600
N	1.18176300	-0.52536900	-0.03639100
N	0.19317800	-1.37691000	-0.03249500
O	-2.09256100	-1.58493100	0.05867800
O	2.09259300	1.58509100	0.05536300
C	-2.53521200	1.08739200	-0.07843500
H	-3.06909500	0.65678000	-0.92649500
H	-3.06542200	0.80268900	0.83183300
H	-2.43823000	2.16546500	-0.16458600
C	2.53520000	-1.08745600	-0.07804300
H	3.06874400	-0.65798900	-0.92689500
H	3.06579900	-0.80157400	0.83163000
H	2.43815500	-2.16564000	-0.16271800

Sum of electronic and zero-point Energies = -525.479959 Hartree

Number of imaginary frequencies: 0

1-planar

	angstroms		
	X	Y	Z
C	-1.25588000	-0.64275700	0.00000000
C	1.25588000	0.64275700	0.00000000
N	-1.18631900	0.72579300	0.00000000
N	-0.01160400	1.29360900	0.00000000
N	0.01160400	-1.29360900	0.00000000
N	1.18631900	-0.72579300	0.00000000
O	2.28857200	1.28940600	0.00000000
O	-2.28857200	-1.28940600	0.00000000
C	0.01160400	2.75957900	0.00000000
H	0.55314500	3.10446600	0.88207500
H	0.55314500	3.10446600	-0.88207500
H	-1.01873700	3.10221900	0.00000000
C	-0.01160400	-2.75957900	0.00000000
H	-0.55314500	-3.10446600	0.88207500
H	-0.55314500	-3.10446600	-0.88207500
H	1.01873700	-3.10221900	0.00000000

Sum of electronic and zero-point Energies = -525.476659 Hartree

Number of imaginary frequencies: 1

1-planar[b]

angstroms

	X	Y	Z
C	-1.246334000	-0.643898000	0.000000000
C	1.246334000	0.643898000	0.000000000
N	-1.176702000	0.716570000	0.000000000
N	-0.011143000	1.283845000	0.000000000
N	0.011143000	-1.283845000	0.000000000
N	1.176702000	-0.716570000	0.000000000
O	2.272494000	1.293403000	0.000000000
O	-2.272494000	-1.293403000	0.000000000
C	0.011143000	2.744100000	0.000000000
H	0.552584000	3.087370000	0.881039000
H	0.552584000	3.087370000	-0.881039000
H	-1.018957000	3.084745000	0.000000000
C	-0.011143000	-2.744100000	0.000000000
H	-0.552584000	-3.087370000	0.881039000
H	-0.552584000	-3.087370000	-0.881039000
H	1.018957000	-3.084745000	0.000000000

Sum of electronic and zero-point Energies = -525.246222 Hartree

Number of imaginary frequencies: 0

Table 2. Structures of compound type **5**

5a

	angstroms		
	X	Y	Z
C	1.16497800	0.88313600	-0.00008700
C	-1.16497300	-0.88314800	0.00008500
N	-1.24500000	0.53499500	-0.00002000
N	1.24500500	-0.53499100	0.00008600
O	-2.18952700	-1.57465400	0.00019800
O	2.18952200	1.57465900	-0.00031700
C	-2.60499900	1.08308900	0.00002300
H	-3.13747200	0.72046500	-0.87958300
H	-3.13666100	0.72261600	0.88102400
H	-2.55213400	2.16949500	-0.00128400
C	2.60500200	-1.08309100	0.00004200
H	3.13646400	-0.72319300	-0.88132000
H	3.13767900	-0.71990000	0.87928600
H	2.55213300	-2.16949500	0.00204200
C	-0.17409900	1.35065500	-0.00000200
H	-0.34741000	2.41685400	-0.00004500
C	0.17409900	-1.35065900	0.00005300
H	0.34742100	-2.41685600	0.00011500

Sum of electronic and zero-point Energies = -493.417313 Hartree

Number of imaginary frequencies: 0

5a[b]

	angstroms		
	X	Y	Z
C	-1.159668000	0.876498000	0.000160000
C	1.159666000	-0.876503000	-0.000169000
N	1.237637000	0.528448000	0.000016000
N	-1.237639000	-0.528445000	-0.000061000
O	2.182619000	-1.563710000	-0.000331000
O	-2.182615000	1.563714000	0.000440000
C	2.593498000	1.072053000	-0.000010000
H	3.123307000	0.706847000	0.878535000
H	3.122627000	0.708677000	-0.879739000
H	2.542989000	2.157686000	0.001104000
C	-2.593500000	-1.072054000	-0.000024000
H	-3.122465000	-0.709129000	0.879993000
H	-3.123473000	-0.706401000	-0.878280000
H	-2.542987000	-2.157685000	-0.001683000
C	0.173396000	1.342120000	0.000103000
H	0.348176000	2.407549000	0.000225000
C	-0.173394000	-1.342122000	-0.000135000
H	-0.348180000	-2.407550000	-0.000256000

Sum of electronic and zero-point Energies = -493.181281 Hartree

Number of imaginary frequencies: 0

5b

	angstroms		
	X	Y	Z
C	1.17558000	0.86863900	-0.00023700
C	-1.17557400	-0.86864300	0.00016700
N	-1.26330600	0.54512800	-0.00001900
N	1.26331200	-0.54512900	0.00006000
O	-2.18425400	-1.56044700	0.00036800
O	2.18425900	1.56044600	-0.00047500
C	-2.64081500	1.06356900	0.00001200
H	-3.15665900	0.68059200	-0.88070800
H	-3.15598300	0.68254300	0.88199100
H	-2.62412000	2.14891000	-0.00115200
C	2.64082100	-1.06357100	0.00008000
H	3.15585200	-0.68306900	-0.88221000
H	3.15680300	-0.68007200	0.88048800
H	2.62412600	-2.14891100	0.00187000
C	-0.17846800	1.36300500	-0.00008600
C	0.17847300	-1.36300700	0.00010300
C	0.35140700	-2.76845000	0.00015500
N	0.50086000	-3.91586000	0.00017000
C	-0.35140000	2.76844800	-0.00009800
N	-0.50085200	3.91585800	-0.00007900

Sum of electronic and zero-point Energies = -677.941116 Hartree

Number of imaginary frequencies: 0

5c

	X	Y	Z
	angstroms		
C	2.14572200	0.00000000	1.18367700
C	-0.79358200	-0.00001800	1.18598200
N	0.00000000	0.00000000	2.37244900
N	1.35209400	-0.00002300	-0.00286100
O	-2.02199100	-0.00002600	1.23256800
O	3.37412700	0.00005100	1.13700800
C	-0.75214700	0.00010200	3.64015900
H	-1.80570900	0.00095300	3.37844200
H	-0.49916000	-0.88988300	4.21604100
H	-0.49778000	0.88926500	4.21668400
C	2.10428000	-0.00000800	-1.27054100
H	1.85146200	0.89013700	-1.84624800
H	3.15784100	-0.00108300	-1.00880900
H	1.84980300	-0.88902100	-1.84724600
C	1.35214500	-0.00007000	2.36960200
C	0.00000000	0.00000000	0.00000000
Cl	-0.85074500	0.00003900	-1.49678500
Cl	2.20282000	-0.00021500	3.86643300

Sum of electronic and zero-point Energies = -1412.668368 Hartree

Number of imaginary frequencies: 0

5d

	angstroms		
	X	Y	Z
C	2.12773500	0.00000000	1.15848300
C	-0.77171900	-0.01830200	1.18885300
N	0.00000000	0.00000000	0.00000000
N	1.35601900	-0.01850700	2.34733300
O	-2.01265000	-0.04246000	1.14389700
O	3.36867300	0.02368100	1.20344400
C	-0.77412800	0.01899800	-1.25054000
H	-0.77331400	-0.97290400	-1.70847900
H	-1.79461800	0.28823400	-0.99296900
H	-0.34249100	0.73961000	-1.94250200
C	2.13015600	-0.03772800	3.59786400
H	3.15064800	-0.30690700	3.34023800
H	2.12934400	0.95409100	4.05598200
H	1.69852700	-0.75846600	4.28969900
C	1.35602000	-0.01853500	-0.04029700
C	0.00000000	0.00000000	2.38763500
C	-0.72847800	0.02119800	3.69256600
H	-0.64581800	-0.93598400	4.22177200
H	-0.35012000	0.80035800	4.36039200
H	-1.78249500	0.19968700	3.48822300
C	2.08450400	-0.04002300	-1.34522000
H	1.70614000	-0.81932000	-2.01288400
H	2.00185700	0.91704800	-1.87462700

H 3.13851900 -0.21847800 -1.14083600

Sum of electronic and zero-point Energies = -572.025043 Hartree

Number of imaginary frequencies: 0

5e

	angstroms		
	X	Y	Z
C	2.15627900	0.00000800	1.19866800
C	-0.80413800	0.00001900	1.17096500
N	-0.00705700	0.00002500	2.35724000
N	1.35917800	-0.00001000	0.01236700
O	-2.03419700	0.00001000	1.20109700
O	3.38633500	0.00004600	1.16849900
C	-0.72902500	0.00006100	3.64311300
H	-1.78833400	0.00088400	3.40173500
H	-0.46415400	-0.89046200	4.21257900
H	-0.46282100	0.88973500	4.21326300
C	2.08115700	-0.00004800	-1.27349900
H	1.81647900	0.89059900	-1.84286400
H	3.14046400	-0.00110400	-1.03211000
H	1.81477000	-0.88959800	-1.84375400
C	1.33593400	-0.00002700	2.35333300
C	0.01620600	0.00004800	0.01627900
F	1.92387700	-0.00007000	3.54479800
F	-0.57175900	0.00010800	-1.17516900

Sum of electronic and zero-point Energies = -691.955899 Hartree

Number of imaginary frequencies: 0

5f

	angstroms		
	X	Y	Z
C	2.11576300	0.02270000	1.18782800
C	-0.76176900	-0.03273200	1.18057800
N	-0.01729200	-0.00242600	2.36837800
N	1.37146800	-0.00743900	0.00026700
O	-2.01162900	-0.05704500	1.13628800
O	3.36563800	0.04765500	1.23251500
C	-0.74613600	0.00086900	3.64685100
H	-1.80570600	-0.01922400	3.40748600
H	-0.46417800	-0.87611700	4.22948200
H	-0.49270800	0.89989700	4.20854300
C	2.10023100	-0.01044100	-1.27826800
H	1.81964800	0.86766600	-1.85989500
H	3.15984900	0.00779900	-1.03897400
H	1.84530400	-0.90840300	-1.84095000
C	1.33113300	0.02327800	2.36505900
C	0.02285600	-0.03311300	0.00356600
O	1.97326100	0.05122400	3.54154200
H	2.92034100	0.06561200	3.29690800
O	-0.61891900	-0.06079200	-1.17322100
H	-1.56602600	-0.07627100	-0.92903100

Sum of electronic and zero-point Energies = -643.925697 Hartree

Number of imaginary frequencies: 0

5f[b]

	angstroms		
	X	Y	Z
C	-0.471870000	1.351010000	0.000426000
C	0.471826000	-1.351065000	0.000291000
N	1.338060000	-0.263629000	0.000149000
N	-1.338039000	0.263668000	0.000069000
O	0.840590000	-2.542094000	-0.000291000
O	-0.840475000	2.542095000	-0.000114000
C	2.780158000	-0.524600000	-0.000044000
H	2.906206000	-1.603060000	0.002972000
H	3.228520000	-0.083766000	-0.888902000
H	3.229646000	-0.078310000	0.885472000
C	-2.780145000	0.524600000	-0.000287000
H	-3.228620000	0.085076000	0.889172000
H	-2.906238000	1.603048000	-0.004785000
H	-3.229494000	0.076972000	-0.885186000
C	0.892216000	1.001619000	0.000081000
C	-0.892172000	-1.001654000	0.000160000
O	1.785299000	1.993102000	-0.000177000
H	1.244196000	2.806469000	0.000134000
O	-1.785411000	-1.993060000	0.000049000
H	-1.244463000	-2.806503000	0.000099000

Sum of electronic and zero-point Energies = -643.652315 Hartree

Number of imaginary frequencies: 0

5g

	angstroms		
	X	Y	Z
C	2.12260800	-0.01830200	1.15238200
C	-0.76693100	-0.03369700	1.21517300
N	0.02334300	0.00079600	2.37759900
N	1.33198300	0.00303100	-0.01024300
O	-2.01541700	-0.05891500	1.25474000
O	3.37130900	-0.03029000	1.11271100
C	-0.68843200	0.04459600	3.65917600
H	-1.73610600	0.23124000	3.43934100
H	-0.59942200	-0.91309800	4.18010700
H	-0.26952200	0.84002300	4.27675200
C	2.04336600	0.04943100	-1.29196100
H	1.61578000	0.83767600	-1.91278900
H	3.08889400	0.24846800	-1.07278400
H	1.96495000	-0.91130600	-1.80897900
C	1.38300900	-0.01379500	2.36495600
C	-0.02736200	-0.02606400	0.00249400
N	2.10343700	0.06468100	3.54088200
H	1.78702600	-0.49616600	4.32028500
H	3.09471700	-0.03722900	3.34528200
N	-0.74870300	0.03982200	-1.17360200
H	-0.42616400	-0.52035000	-1.95097400
H	-1.73882700	-0.07206300	-0.97756300

Sum of electronic and zero-point Energies = -604.152609 Hartree

Number of imaginary frequencies: 0

5h-chair

	angstroms		
	X	Y	Z
C	1.15635800	0.87211500	0.10047000
C	-1.15206500	-0.87186400	-0.09138600
N	-1.28961700	0.53504700	0.05232100
N	1.29384300	-0.53456600	-0.04273200
O	-2.11648400	-1.59104700	-0.25955000
O	2.12066200	1.59170900	0.26792700
C	-2.67390600	1.03359800	0.17891300
H	-2.98876000	1.52202000	-0.74486200
H	-3.29654900	0.15970400	0.35706300
H	-2.73195900	1.74666200	0.99533800
C	2.67801100	-1.03326800	-0.16973700
H	3.30037300	-0.15979000	-0.35099100
H	2.99415800	-1.51912100	0.75494800
H	2.73510600	-1.74849600	-0.98432200
C	-0.21337100	1.36329700	-0.00804600
C	0.21752600	-1.36299800	0.01792300
N	-0.25504700	2.70238600	-0.40555000
O	-1.31619700	3.30904300	-0.37859400
N	0.25909900	-2.70169900	0.41637600
O	1.32080200	-3.30760500	0.39203400

Sum of electronic and zero-point Energies = -752.060075 Hartree

Number of imaginary frequencies: 0

5h-planar

	angstroms		
	X	Y	Z
C	-1.41215500	0.33553100	0.00000000
C	1.41215500	-0.33553100	0.00000000
N	0.94596500	1.02246900	0.00000000
N	-0.94596500	-1.02246900	0.00000000
O	2.60587700	-0.55090300	0.00000000
O	-2.60587700	0.55090300	0.00000000
C	2.07321000	1.98489600	0.00000000
H	2.68560400	1.77833400	0.87836200
H	2.68560400	1.77833400	-0.87836200
H	1.69838800	2.99590100	0.00000000
C	-2.07321000	-1.98489600	0.00000000
H	-2.68560400	-1.77833400	0.87836200
H	-2.68560400	-1.77833400	-0.87836200
H	-1.69838800	-2.99590100	0.00000000
C	-0.36327400	1.35502900	0.00000000
C	0.36327400	-1.35502900	0.00000000
N	-0.94596500	2.65697800	0.00000000
O	-0.20521000	3.63033200	0.00000000
N	0.94596500	-2.65697800	0.00000000
O	0.20521000	-3.63033200	0.00000000

Sum of electronic and zero-point Energies = -752.049581 Hartree

Number of imaginary frequencies: 5

5h-planar[b]

	angstroms		
	X	Y	Z
C	-1.406738000	0.330400000	0.000000000
C	1.406738000	-0.330400000	0.000000000
N	0.941642000	1.012862000	0.000000000
N	-0.941642000	-1.012862000	0.000000000
O	2.598927000	-0.545789000	0.000000000
O	-2.598927000	0.545789000	0.000000000
C	2.065456000	1.969136000	0.000000000
H	2.676971000	1.758141000	0.876471000
H	2.676971000	1.758141000	-0.876471000
H	1.699511000	2.981981000	0.000000000
C	-2.065456000	-1.969136000	0.000000000
H	-2.676971000	-1.758141000	0.876471000
H	-2.676971000	-1.758141000	-0.876471000
H	-1.699511000	-2.981981000	0.000000000
C	-0.364073000	1.338016000	0.000000000
C	0.364073000	-1.338016000	0.000000000
N	-0.941642000	2.640707000	0.000000000
O	-0.204612000	3.599960000	0.000000000
N	0.941642000	-2.640707000	0.000000000
O	0.204612000	-3.599960000	0.000000000

Sum of electronic and zero-point Energies = -751.724680 Hartree

Number of imaginary frequencies: 5

5i

	angstroms		
	X	Y	Z
C	1.17428300	0.86887600	-0.03946900
C	-1.17398700	-0.86877600	-0.04885100
N	-1.24472400	0.56164200	-0.05123900
N	1.24504200	-0.56155500	-0.04471100
O	-2.20535000	-1.52745400	-0.11300600
O	2.20601400	1.52767700	-0.09571900
C	-2.63317700	1.06108400	-0.16823100
H	-3.16672400	0.35539600	-0.79962300
H	-3.10490200	1.06519700	0.81508000
H	-2.64301600	2.05404300	-0.59938900
C	2.63419600	-1.06075900	-0.15416600
H	3.17175800	-0.35342400	-0.78028500
H	3.09965900	-1.06751900	0.83210600
H	2.64672900	-2.05256400	-0.58792400
C	-0.16600300	1.37370100	0.02956400
C	0.16582800	-1.37376900	0.02745900
C	-0.38632700	2.87111900	0.18652500
C	0.38515100	-2.87152000	0.18246000
F	0.69511200	3.52989200	0.57388400
F	-0.81555600	3.43024400	-0.97692400
F	-1.34945100	3.11559100	1.11489900
F	1.34236000	-3.11802100	1.11640800
F	0.82187300	-3.42797500	-0.97955400
F	-0.69871800	-3.53114900	0.56136100

Sum of electronic and zero-point Energies = -1167.676519 Hartree

Number of imaginary frequencies: 0

5j

	angstroms		
	X	Y	Z
C	1.16811100	0.89203700	0.13090000
C	-1.16807600	-0.89207700	-0.13143000
N	-1.26540500	0.53337900	-0.06442600
N	1.26542500	-0.53339300	0.06435900
O	-2.15308600	-1.60004700	-0.27688700
O	2.15311300	1.60015400	0.27582000
C	-2.63284300	1.08532400	-0.17156800
H	-3.25607700	0.29615000	-0.58192300
H	-2.98880500	1.36811300	0.81876200
H	-2.62455300	1.95516500	-0.82610800
C	2.63282100	-1.08527000	0.17206300
H	2.98920600	-1.36824300	-0.81806800
H	3.25589400	-0.29599700	0.58247100
H	2.62431400	-1.95503500	0.82669900
C	-0.19085000	1.32794700	0.06324500
C	0.19086600	-1.32804700	-0.06335000
N	-0.42224500	2.77510000	0.18609100
O	-1.05344000	3.14705200	1.16640300
O	0.03044600	3.47803700	-0.69700000
N	0.42226700	-2.77511900	-0.18610300
O	-0.03191600	-3.47820000	0.69613500

O	1.05490800	-3.14705100	-1.16553300

Sum of electronic and zero-point Energies = -902.500813 Hartree

Number of imaginary frequencies: 0

5k-boat

	angstroms		
	X	Y	Z
C	-1.29579200	0.65073300	-0.19273400
C	1.26715600	-0.75349000	-0.16509300
N	1.15640300	0.67766700	-0.11229900
N	-1.18611300	-0.77972700	-0.12716800
O	2.35342500	-1.28863200	-0.30413500
O	-2.37928500	1.18503200	-0.35481400
C	2.46444300	1.37044300	-0.18797600
H	2.88593700	1.45785900	0.81364100
H	3.11299700	0.74185500	-0.79187700
H	2.33930100	2.34875200	-0.63861500
C	-2.49265800	-1.47323600	-0.22060500
H	-3.13054900	-0.84922800	-0.84039900
H	-2.35964900	-2.45482100	-0.66177200
H	-2.93158700	-1.55335500	0.77411000
C	-0.03051600	1.31235200	-0.04064100
C	-0.00054700	-1.41393200	-0.03008100
S	-0.11150700	3.10395800	0.21219600
O	-1.33247500	3.49523000	0.84848900
O	0.45694300	3.79066300	-0.92251000
S	0.07553600	-3.20340200	0.23789500

O	-0.47379900	-3.89894600	-0.90081400
O	1.28541900	-3.58997200	0.89778600
F	1.03653200	3.17301300	1.39036600
F	-1.09233300	-3.26292000	1.39686300

Sum of electronic and zero-point Energies = -1789.197965 Hartree

Number of imaginary frequencies: 0

5k-planar

	angstroms		
	X	Y	Z
C	-1.26618400	0.66938500	0.00051400
C	1.31485000	-0.76241700	0.00058800
N	1.19116000	0.67274500	0.00029500
N	-1.14241500	-0.76733300	0.00017400
O	2.41607400	-1.28617300	0.00074900
O	-2.36794600	1.19178000	0.00117100
C	2.49186900	1.38854300	0.00023800
H	2.56425000	2.00687000	0.89353800
H	3.26368100	0.62652700	0.00155500
H	2.56528800	2.00482300	-0.89439400
C	-2.44302800	-1.48306500	0.00001600
H	-3.21476800	-0.72097700	0.00038300
H	-2.51599400	-2.10007500	-0.89416800
H	-2.51585900	-2.10070900	0.89378500
C	0.00907300	1.32527100	0.00008800
C	0.03944500	-1.41943400	0.00013300
S	0.12720400	3.14427900	-0.00050500

O	0.67698700	3.57315200	-1.26328000
O	0.67523600	3.57429600	1.26264100
S	-0.07850200	-3.23888200	-0.00024200
O	-0.62653500	-3.66843900	1.26283600
O	-0.62848500	-3.66777100	-1.26270200
F	-1.40678200	3.58172900	-0.00181600
F	1.45520200	-3.67589800	-0.00161600

Sum of electronic and zero-point Energies = -1789.187972 Hartree

Number of imaginary frequencies: 3

5l-boat

	angstroms		
	X	Y	Z
C	-1.23862300	0.74454600	0.06169800
C	1.23780600	-0.74324700	0.07794300
N	1.20995400	0.67437400	-0.02114100
N	-1.21012200	-0.67405300	-0.02318600
O	2.30006600	-1.34778000	0.19447300
O	-2.30170100	1.35006600	0.16483800
C	2.53745600	1.32338500	-0.09846000
H	2.73855100	1.86599100	0.82466000
H	3.26340900	0.52761500	-0.23009300
H	2.55092200	2.02944900	-0.92357000
C	-2.53709000	-1.32374900	-0.10368700
H	-3.26190300	-0.52936000	-0.24924100
H	-2.54435300	-2.03864800	-0.92120500
H	-2.74534000	-1.85636800	0.82368100

C	0.05676600	1.38466400	-0.01260500
C	-0.05711200	-1.38415900	0.00092600
O	0.98628600	3.60002900	-0.05134700
O	-0.98645400	-3.59982500	-0.02218700
C	0.01791700	2.86524600	-0.06678200
H	-1.00888400	3.25238100	-0.11992100
C	-0.01793400	-2.86524500	-0.03768400
H	1.00922600	-3.25287800	-0.07874000

Sum of electronic and zero-point Energies = -720.110090 Hartree

Number of imaginary frequencies: 0

5l-planar

	angstroms		
	X	Y	Z
C	-1.24188900	0.74650100	0.00000000
C	1.24188900	-0.74650200	0.00000000
N	1.21080800	0.67542300	0.00000000
N	-1.21080800	-0.67542300	0.00000000
O	2.30745400	-1.35638000	0.00000000
O	-2.30745400	1.35638000	0.00000000
C	2.53719100	1.33273500	0.00000000
H	2.63507500	1.96146000	0.88239500
H	3.27517500	0.53770300	0.00000000
H	2.63507500	1.96146000	-0.88239500
C	-2.53719100	-1.33273500	0.00000000
H	-3.27517400	-0.53770300	0.00000000

H	-2.63507500	-1.96146000	-0.88239500
H	-2.63507500	-1.96146000	0.88239500
C	0.05697900	1.38503000	0.00000000
C	-0.05697900	-1.38503000	0.00000000
O	0.99224900	3.59884500	0.00000000
O	-0.99224900	-3.59884500	0.00000000
C	0.02158600	2.86691100	0.00000000
H	-1.00517900	3.25768300	0.00000000
C	-0.02158600	-2.86691100	0.00000000
H	1.00517900	-3.25768300	0.00000000

Sum of electronic and zero-point Energies = -720.106792 Hartree

Number of imaginary frequencies: 2

Table 3. Structures of compound type **6**

6a

	angstroms		
	X	Y	Z
C	1.14597900	0.87958500	-0.00032900
C	-1.14597600	-0.87958800	0.00028800
N	-1.25553300	0.52428600	-0.00000200
N	1.25553500	-0.52428700	0.00003900
C	-2.60152500	1.10908100	0.00002000
H	-3.14242400	0.75913800	-0.87978900
H	-3.14196100	0.76023800	0.88055600
H	-2.51324200	2.19332900	-0.00066600
C	2.60152800	-1.10908400	0.00006200
H	3.14189200	-0.76050600	-0.88062500
H	3.14249900	-0.75887400	0.87971900
H	2.51324500	-2.19333000	0.00107400
C	-0.18239300	1.32964300	-0.00022900
H	-0.36183900	2.39425400	-0.00040300
C	0.18239600	-1.32964500	0.00025100
H	0.36184200	-2.39425600	0.00045500
S	2.48092600	1.91939000	-0.00088500
S	-2.48092400	-1.91939100	0.00072900

Sum of electronic and zero-point Energies = -1139.352549 Hartree

Number of imaginary frequencies: 0

6b

	angstroms		
	X	Y	Z
C	1.16054000	0.87756000	-0.00036900
C	-1.16053700	-0.87756200	0.00036100
N	-1.25551100	0.53432600	0.00001900
N	1.25551400	-0.53432700	0.00001600
C	-2.61076200	1.11404800	0.00001700
H	-3.13926900	0.74865100	-0.88045700
H	-3.13889900	0.74961000	0.88111500
H	-2.54320000	2.19576500	-0.00057200
C	2.61076400	-1.11405000	0.00005400
H	3.13885500	-0.74981000	-0.88115500
H	3.13931900	-0.74845500	0.88041600
H	2.54320300	-2.19576700	0.00088000
C	-0.17046500	1.32206400	-0.00028200
C	0.17046800	-1.32206600	0.00029700
S	2.49739900	1.90087800	-0.00088900
S	-2.49739600	-1.90088000	0.00081000
F	-0.40147100	2.62324300	-0.00054400
F	0.40147400	-2.62324500	0.00055500

Sum of electronic and zero-point Energies = -1337.889730 Hartree

Number of imaginary frequencies: 0

6c-chair

	angstroms		
	X	Y	Z
C	1.14964400	0.83743700	0.14709100
C	-1.12466600	-0.83323100	-0.11883700
N	-1.28275200	0.52916800	0.07437500
N	1.30777300	-0.52481800	-0.04580800
C	-2.64632500	1.07337200	0.22948300
H	-3.00428100	1.48887600	-0.71378500
H	-3.28484900	0.23386900	0.50776400
H	-2.63916400	1.84633400	0.99004500
C	2.67142300	-1.06886200	-0.20090400
H	3.30961000	-0.22946800	-0.48032700
H	3.02986300	-1.48322200	0.74268700
H	2.66412400	-1.84263900	-0.96060800
C	-0.18600800	1.34677700	0.04353300
C	0.21099900	-1.34259600	-0.01500100
S	2.38376700	1.90978300	0.49108000
S	-2.35886400	-1.90517400	-0.46349100
N	-0.21157300	2.67619100	-0.37809600
O	-1.21294900	3.36159200	-0.25706100
N	0.23660000	-2.67192100	0.40686600
O	1.23846800	-3.35690000	0.28710300

Sum of electronic and zero-point Energies = -1397.989630 Hartree

Number of imaginary frequencies: 0

6c-planar

	angstroms		
	X	Y	Z
C	-0.72042700	1.25968700	0.00000000
C	0.72042700	-1.25968700	0.00000000
N	1.38718500	0.00559500	0.00000000
N	-1.38718500	-0.00559500	0.00000000
C	2.86363600	-0.09065800	0.00000000
H	3.15789300	-0.67035600	0.87600400
H	3.15789300	-0.67035600	-0.87600400
H	3.30363100	0.89189700	0.00000000
C	-2.86363600	0.09065800	0.00000000
H	-3.15789300	0.67035600	0.87600400
H	-3.15789300	0.67035600	-0.87600400
H	-3.30363100	-0.89189700	0.00000000
C	0.72042700	1.18036200	0.00000000
C	-0.72042700	-1.18036200	0.00000000
N	1.29970600	2.51987300	0.00000000
O	2.50522300	2.65021700	0.00000000
N	-1.29970600	-2.51987300	0.00000000
O	-2.50522300	-2.65021700	0.00000000
S	1.58959500	-2.68455200	0.00000000
S	-1.58959500	2.68455200	0.00000000

Sum of electronic and zero-point Energies = -1397.956854 Hartree

Number of imaginary frequencies: 5

6d-boat

	angstroms		
	X	Y	Z
C	-1.26113300	0.63140400	-0.33549900
C	1.23489100	-0.73511400	-0.30876800
N	1.16269300	0.67558200	-0.18903300
N	-1.19114800	-0.77826700	-0.20389600
C	2.44156900	1.40194300	-0.36493000
H	3.12342000	1.11363800	0.43522000
H	2.86479100	1.07934800	-1.31488500
H	2.27329800	2.46988500	-0.37516200
C	-2.46682100	-1.50599100	-0.39661700
H	-2.87318200	-1.19089800	-1.35640800
H	-2.29856300	-2.57398100	-0.39547900
H	-3.16263800	-1.21139100	0.38908300
C	-0.03283700	1.28373600	-0.05257600
C	0.00184100	-1.38534500	-0.04223400
S	-0.12596600	3.01078000	0.53121700
O	-1.27870200	3.23243500	1.35275200
O	0.34002600	3.92330500	-0.48157100
S	0.08470800	-3.10786000	0.55616900
O	-0.36374200	-4.02808400	-0.45757600
O	1.22303100	-3.32323500	1.39919600
F	1.11365600	2.86905700	1.60660800
F	-1.17334100	-2.95806300	1.60883600
S	-2.63114000	1.44684700	-0.83366000
S	2.61336800	-1.55408100	-0.77682500

Sum of electronic and zero-point Energies = -2435.111661 Hartree

Number of imaginary frequencies: 0

6d-planar

	angstroms		
	X	Y	Z
C	-1.22753600	0.80859000	0.00000000
C	1.22753600	-0.80859000	0.00000000
N	1.21553900	0.63428400	0.00000000
N	-1.21553900	-0.63428400	0.00000000
C	2.54913800	1.29600700	0.00000000
H	2.64930600	1.90305700	0.89631200
H	3.29127800	0.50551000	0.00000000
H	2.64930600	1.90305700	-0.89631200
C	-2.54913800	-1.29600700	0.00000000
H	-3.29127800	-0.50551000	0.00000000
H	-2.64930600	-1.90305700	-0.89631200
H	-2.64930600	-1.90305700	0.89631200
C	0.07877000	1.35965300	0.00000000
C	-0.07877000	-1.35965300	0.00000000
S	0.41065100	3.20012400	0.00000000
O	1.00969300	3.53146500	-1.26911100
O	1.00969300	3.53146500	1.26911100
S	-0.41065100	-3.20012400	0.00000000
O	-1.00969300	-3.53146500	1.26911100
O	-1.00969300	-3.53146500	-1.26911100
F	-1.00969300	3.92032000	0.00000000

F	1.00969300	-3.92032000	0.00000000
S	-2.66455500	1.67323700	0.00000000
S	2.66455500	-1.67323700	0.00000000

Sum of electronic and zero-point Energies = -2435.088639 Hartree

Number of imaginary frequencies: 3

Table 4. Structures of compound type **7**

7-boat

	angstroms		
	X	Y	Z
C	1.05806200	0.88146300	0.21842800
C	-1.05792100	-0.88107100	0.22026100
N	-0.19575400	1.34914400	0.00635100
N	-1.18838700	0.50445600	-0.02566000
N	1.18840000	-0.50443500	-0.02552300
N	0.19578100	-1.34906400	0.00818400
C	-2.51495100	1.08392100	-0.26107600
H	-3.07538700	0.40918600	-0.90840300
H	-3.04821900	1.17030200	0.68657500
H	-2.36168100	2.06111600	-0.71155000
C	2.51485200	-1.08425700	-0.26069300
H	3.07498100	-0.41048800	-0.90929100
H	3.04856800	-1.16921800	0.68683500
H	2.36136700	-2.06212400	-0.70963200
S	-2.33014200	-1.86092900	0.66666400
S	2.33046100	1.86190900	0.66302900

Sum of electronic and zero-point Energies = -1171.399368 Hartree

Number of imaginary frequencies: 0

7-planar

	angstroms		
	X	Y	Z
C	-1.25673800	-0.62486800	0.00000000
C	1.25673800	0.62486800	0.00000000
N	-1.15880500	0.72665100	0.00000000
N	0.00670200	1.30016100	0.00000000
N	-0.00670200	-1.30016100	0.00000000
N	1.15880500	-0.72665100	0.00000000
C	-0.00670200	2.76546500	0.00000000
H	0.52828000	3.12265900	0.88160400
H	0.52828000	3.12265900	-0.88160400
H	-1.04768000	3.07566400	0.00000000
C	0.00670200	-2.76546500	0.00000000
H	-0.52828000	-3.12265900	0.88160400
H	-0.52828000	-3.12265900	-0.88160400
H	1.04768000	-3.07566400	0.00000000
S	2.72693800	1.41729700	0.00000000
S	-2.72693800	-1.41729700	0.00000000

Sum of electronic and zero-point Energies = -1171.396573 Hartree

Number of imaginary frequencies: 1
